# Breast magnetic resonance imaging for surveillance of women with a personal history of breast cancer: outcomes stratified by interval between definitive surgery and surveillance MR imaging

**DOI:** 10.1186/s12885-018-3998-1

**Published:** 2018-01-22

**Authors:** Vivian Youngjean Park, Eun-Kyung Kim, Min Jung Kim, Hee Jung Moon, Jung Hyun Yoon

**Affiliations:** Department of Radiology and Research Institute of Radiological Science, Severance Hospital, Yonsei University College of Medicine, 50-1 Yonsei-ro, Seodaemun-gu, 03722 Seoul, Republic of Korea

**Keywords:** Breast cancer, Surveillance, Magnetic resonance imaging

## Abstract

**Background:**

Women with a personal history of breast cancer are at increased risk of future breast cancer events, and may benefit from supplemental screening methods that could enhance early detection of subclinical disease. However, current literature on breast magnetic resonance (MR) imaging surveillance is limited. We investigated outcomes of surveillance breast magnetic resonance (MR) imaging in women with a personal history of breast cancer.

**Methods:**

We reviewed 1053 consecutive breast MR examinations that were performed for surveillance in 1044 women (median age, 53 years; range, 20–85 years) previously treated for breast cancer between August 2014 and February 2016. All patients had previously received supplemental surveillance with ultrasound. Cancer detection rate (CDR), abnormal interpretation rate and characteristics of MR-detected cancers were assessed, including extramammary cancers. We also calculated the PPV_**1**_, PPV_**3**_, sensitivity and specificity for MR-detected intramammary lesions. Performance statistics were stratified by interval following initial surgery.

**Results:**

The CDR for MR-detected cancers was 6.7 per 1000 examinations (7 of 1053) and was 3.8 per 1000 examinations (4 of 1053) for intramammary cancers. The overall abnormal interpretation rate was 8.0%, and the abnormal interpretation rate for intramammary lesions was 7.2%. The PPV_1_, PPV_3_, sensitivity and specificity for intramammary lesions was 5.3% (4 of 76), 15.8% (3 of 19), 75.0% (3 of 4) and 98.3% (1031 of 1049), respectively. For MR examinations performed ≤36 months after surgery, the overall CDR was 1.4 per 1000 examinations. For MR examinations performed > 36 months after surgery, the overall CDR was 17.4 per 1000 examinations.

**Conclusions:**

Surveillance breast MR imaging may be considered in women with a history of breast cancer, considering the low abnormal interpretation rate and its high specificity. However, the cancer detection rate was low and implementation may be more effective after more than 3 years after surgery.

## Background

Although women previously treated for breast cancer are at a statistically significant increased risk of future breast cancer events [1, 2], annual mammographic screening is currently the only post-treatment imaging modality recommended for breast cancer follow-up by the American Society for Clinical Oncology (ASCO) and the National Comprehensive Cancer Network (NCCN) [3, 4].

There has been limited information on breast magnetic resonance (MR) imaging surveillance in this specific patient population. Previous screening trials using MR imaging have focused on high-risk women without a personal history of breast cancer, resulting in increased invasive breast cancer yields at acceptable recall rates and positive predictive values (PPV) of biopsy [5–8]. Surveillance breast MR imaging may also have potential benefits in women previously treated for breast cancer, primarily by overcoming the decreased sensitivity of mammography in breasts with dense tissue and treatment-related changes [9–11]. Despite advances in locoregional and systemic therapy, recurrence or second breast cancer rates are approximately 3% to 5% per year even in early-stage hormone receptor-positive patients [2, 12]. Therefore, this patient group would benefit from supplemental screening methods that could enhance early detection of subclinical disease and ultimately improve relative survival [13, 14]. Previous studies have reported that breast MR imaging depicted additional cancers even after prior or concurrent negative findings of mammography and ultrasound (US) [11, 15, 16]. However, due to sparse data on surveillance breast MR imaging, the appropriate interval following surgery for initiation of MRI surveillance has not yet been investigated.

At our institution, surveillance breast MR imaging has recently been implemented as part of the routine post-treatment surveillance protocol for patients previously treated for breast cancer. These patients had previously undergone routine supplemental surveillance with US. As a result, we were able to obtain data from a large group of patients regarding its performance stratified by interval between definitive surgery and implementation of MRI surveillance. The purpose of this study was to investigate the outcomes of surveillance breast MR imaging in women with a personal history of breast cancer.

## Methods

### Study population

**Fig. 1:**
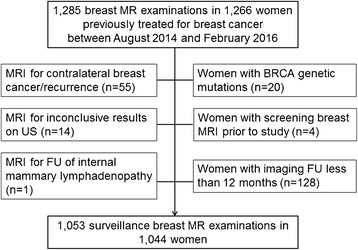
Flowchart of study population selection

This retrospective study was approved by the institutional review board of Yonsei University College of Medicine and the requirement for informed consent was waived. Between August 2014 and February 2016, 1285 breast MR examinations were performed in 1266 women who had been previously treated for breast cancer, either with breast conserving surgery (BCS) (*n* = 648) or mastectomy (*n* = 396). Among them, 222 women were excluded either because they underwent MR imaging for reasons other than postoperative surveillance (*n* = 70); they had BRCA genetic mutations (*n* = 20); they had undergone screening breast MR imaging prior to the study period (*n* = 4); or their 12-month imaging follow-up information was unavailable (*n* = 128) (Fig. 1). Finally, 1053 breast MR examinations that were performed for surveillance in 1044 women (median age, 53 years; range, 20–85 years) with a personal history of breast cancer composed our study population. Among them, 9 women underwent two rounds of screening MR examinations during the study period. Analysis of Breast Imaging Reporting and Data System (BI-RADS) category assessments of mammograms and US performed prior to MR examinations revealed BI-RADS category 1 in 373 examinations (35.4%), category 2 in 434 examinations (41.2%), and category 3 in 246 examinations (23.4%). The median follow-up period after surveillance breast MR imaging was 18.7 months (range, 12.0–30.7 months).

### Post-treatment surveillance

After definitive breast cancer surgery, patients underwent follow-up by clinical examination and breast US every 6 months and with mammography, chest radiography, abdominal US and whole body bone scan every 12 months. After 5 years following initial surgery, patients underwent annual follow-up by breast US and mammography. Breast MR imaging was implemented as part of the routine post-treatment surveillance protocol in 2013, and thereafter patients underwent screening breast MR imaging instead of US at approximately two and five years after surgery. Surveillance breast MR imaging was also performed at the request of clinicians or patients.

The median interval between prior surveillance US and MR examinations was 6.1 months (range, 0–13.9 months). In 19 cases (1.8%), surveillance US and MR imaging were performed on the same day at the request of the referring physician. The median interval between prior mammography and MR examinations was 11.5 months (range, 0–65.1 months).

### MR imaging technique

Breast MR examinations were performed using two 3 − Tesla MR scanners (Discover 750, GE Medical Systems, Milwaukee, WI, USA; Ingenia, Philips Medical Systems, Best, The Netherlands). Imaging was performed with a dedicated phased array breast coil (8-channel GE or 16-channel Philips) with the patient in the prone position. Imaging was performed prior to a rapid bolus injection of contrast agent and six times after injection. Sequences included a three-plane localizing sequence, axial T2-weighted fast-spin-echo and T2-stimulated inversion recovery (STIR) sequence, and axial T1-weighted non-fat-suppressed or fat-suppressed sequence before contrast administration. The bolus injection consisted of 0.2 mmol/kg body weight of gadolinium-based contrast agent (Dotarem, Guerbet, Paris, France; Magnevist, Berlex Laboratories, Wayne, NJ, USA; or Gadovist, Bayer Scherming Pharma, AG, Berlin, Germancy) and a 20-mL saline flush delivered at a rate of 2 ml/s. 3D dynamic post-contrast enhanced (DCE) axial images are then performed in the axial plane and a T1-weighted 3D delayed postcontrast sequence is acquired in the sagittal plane. Bilateral examinations were performed for all patients.

### MR imaging evaluation

MR images were prospectively interpreted by one of four radiologists with 6–15 years of experience in breast MR imaging interpretation. Computer aided evaluation software (CADstream, Confirma, Kirkland, WA) was used for characterization of lesion kinetics. Of the 1044 women, 89.1% (930 of 1044) previously underwent preoperative MR imaging. Each MR examination was given a Breast Imaging Reporting and Data System (BI-RADS) final assessment category based on the breast lesion morphology and kinetics.

We retrospectively reviewed MR imaging reports, clinical and imaging records. For lesions assessed as BI-RADS 3, follow-up breast MRI or US at 6–12 months was recommended based on the presence of a US-correlate on previous imaging and the period of stability. BI-RADS category 3 was also given to newly found lesions if its findings were probably benign according to the BI-RADS MR lexicon [17]. For breast or chest wall lesions that were assessed as BI-RADS category 4 or 5, targeted US was first performed and US-guided biopsy or MR-guided biopsy was performed accordingly. For suspicious extramammary findings found on breast MR imaging, further evaluation with other imaging modalities was performed with subsequent biopsy when needed.

### Statistical analysis

Intramammary cancer was defined as cancer in the ipsilateral breast following BCS or cancer in the contralateral breast. Extramammary cancer was defined as locoregional disease (cancer in the ipsilateral axilla, internal mammary or supraclavicular lymph nodes or in the mastectomy bed) and distant metastasis.

We calculated the overall cancer detection rate and abnormal interpretation rates. The overall cancer detection rate for MRI was defined as the total number of intramammary and extramammary cancers detected at MR imaging per 1000 examinations. The overall abnormal interpretation rate for MRI was defined as the percentage of MR examinations that were given BI-RADS categories 0, 3, 4, 5 or those with findings suspicious for extramammary cancer detected at MR imaging. According to the outcome monitoring section of the BI-RADS 5th edition atlas, we included BI-RADS category 3 in the numerator of the abnormal interpretation rate because further imaging is recommended before the next routine screening [18].

We also calculated outcome measures for MR-detected intramammary lesions. The cancer detection rate for intramammary lesions was defined as the total number of intramammary cancers detected at MR imaging per 1000 examinations. The abnormal interpretation rate for intramammary lesions was defined as the percentage of MR examinations that were given BI-RADS categories 0, 3, 4, or 5. Positive MR examinations were defined as those given BI-RADS categories 4 or 5. Negative MR examinations were defined as those assessed as BI-RADS categories 1, 2 or 3. MR examinations with an initial BI-RADS category 0 assessment were reclassified according to their final assessment. PPV_1_ was defined as the percentage of MR examinations with BI-RADS categories 0, 3, 4, 5 that resulted in a tissue diagnosis of cancer. PPV_3_ was defined as the percentage of all known breast biopsies performed as a result of positive MR examinations that resulted in a tissue diagnosis of cancer. A true-positive (TP) result was defined as a positive MR examination resulting in a diagnosis of cancer within 1 year. A true-negative (TN) result was defined as a negative MR examination and no detection of cancer within 1 year. A false-negative (FN) result was defined as a negative MR examination with a diagnosis of cancer within 1 year. A false-positive (FP) result was defined as a positive MR examination with no detection of cancer within 1 year.

In addition, we stratified the above performance statistics according to the interval between initial surgery and surveillance MR imaging: (1) for MR examinations performed at or less than a 36-month interval and (2) for examinations performed at more than a 36-month interval following initial surgery. Performance statistics were compared between the two groups using the Fisher exact test. We also compared the intervals between prior surveillance US and MR examinations by using the Student *t* test. Statistical analyses were performed by using statistical software (SPSS version 23.0; IBM Corp, Armonk, NY.).

## Results

Table 1 shows the clinical-pathologic characteristics of the 1044 women who underwent screening breast MR examinations. The median interval between initial surgery for breast cancer and first-time screening MR examination was 27.8 months (range, 12.1–167.3 months). The final assessment categories of the 1053 examinations were as follows: BI-RADS category 1 in 545 examinations (51.8%), BI-RADS category 2 in 432 examinations (41.0%), BI-RADS category 3 in 54 (5.1%), BI-RADS category 4 in 21 examinations (2.0%), BI-RADS category 0 in 1 examination (0.1%). Three examinations assigned as BI-RADS category 2 and five examinations assigned as BI-RADS category 1 showed extramammary findings suspicious for malignancy (0.8%, 8 of 1053).

**Table 1:** Characteristics of 1044 women with a personal history of breast cancer

Characteristic	
Age (years)^a^	53 (20–85)
Interval between initial surgery and screening MRI^a^ (months)	27.8 (12.1–167.3)
Preoperative breast MRI	
Yes	930 (89.1%)
No	114 (10.9%)
Pathology of initial breast cancer	
Ductal carcinoma in situ	185 (17.7%)
Invasive ductal carcinoma	729 (69.8%)
Invasive lobular carcinoma	33 (3.2%)
Tubular carcinoma	25 (2.4%)
Cribiform carcinoma	3 (0.3%)
Mucinous carcinoma	23 (2.2%)
Invasive micropapillary carcinoma	6 (0.6%)
Metaplastic carcinoma	6 (0.6%)
Solid papillary carcinoma	10 (1.0%)
Others	24 (2.3%)
Type of surgery	
Partial mastectomy	648 (62.1%)
Mastectomy	396 (37.9%)
Pathological T stage	
TX	15 (1.4%)
T0	27 (2.6%)
Tis	188 (18.0%)
T1	624 (59.8%)
T2	176 (16.9%)
T3	12 (1.1%)
T4	2 (0.2%)
Pathological N stage	
NX	7 (0.7%)
N0	822 (78.7%)
N1	182 (17.4%)
N2	27 (2.6%)
N3	6 (0.6%)

^a^Median value is shown with range in parentheses

### Cancer detection yield for MRI

The overall abnormal interpretation rate for MRI was 8.0% (84 of 1053) and biopsy or further imaging was recommended for 29 examinations (2.7%) with 21 of the 29 exams classified as BI-RADS category 4 and the other 8 exams demonstrating extramammary lesions suspicious for malignancy (Table 2). Of the 21 BI-RADS category 4 lesions, 18 lesions underwent image-guided biopsy (US-guided biopsy [*n* = 16] or MR-guided biopsy [*n* = 2]) and one lesion underwent surgical excision for a US correlate. Among them, 3 lesions were diagnosed as cancer. All three detected cancers were newly developed contralateral breast cancer, with one cancer detected at a second-round MR examination. Of the 54 lesions that were BI-RADS category 3, one cancer was diagnosed (1.8%). This lesion was an 8-mm enhancing mass at the contralateral breast and moderate background parenchymal enhancement on preoperative MR imaging performed 5 years ago made accurate comparison difficult. Because it was considered to have slightly increased in size, ultrasound correlation was recommended and the final assessment was upgraded to BI-RADS category 4 at US. Subsequent US-guided biopsy yielded invasive ductal carcinoma (Table 3). All of the four MR-detected intramammary cancers were not detected on prior surveillance US which was performed at a median interval of 5.5 months (range, 4.6–12.4 months).

**Table 2:** Performance of surveillance breast MR imaging

Performance Statistics	Total (*n* = 1053)	Initial surgery-MR interval ≤ 36 months (*n* = 709)	Initial surgery-MR interval > 36 months (*n* = 344)	*p* value
Cancer detection rate for MRI^a^	6.7 (7/1053)	1.4 (1/709)	17.4 (6/344)	0.006
Abnormal interpretation rate for MRI	84/1053 (8.0%)^c^	51/709 (7.2%)	33/344 (9.6%)	0.184
Cancer detection rate for intramammary lesions^b^	3.8 (4/1053)	1.41 (1/709)	8.7 (3/344)	0.105
Abnormal interpretation rate for intramammary lesions	76/1053 (7.2%)	49/709 (6.9%)	27/344 (7.8%)	0.612
PPV_1_	4/76 (5.3%)	1/49 (2.0%)	3/27 (11.1%)	0.125
PPV_3_	3/19 (15.8%)	1/10 (10.0%)	2/9 (22.2%)	0.582
Sensitivity	3/4 (75.0%)	1/1 (100%)	2/3 (66.7%)	> 0.999
Specificity	1031/1049 (98.3%)	698/708 (98.6%)	333/341 (97.6%)	0.199

^a^Cancer detection rate for MRI is total number of intramammary and extramammary cancers detected at MR imaging per 1000 examinations

^b^Cancer detection rate for intramammary lesions is total number of total number of intramammary cancers detected at MR imaging per 1000 examinations

^c^Percentage is shown in parentheses

**Table 3:** Clinical and Imaging Characteristics of the Four Intramammary Breast Cancers Detected on Surveillance Breast MRI

Age range, years	Initial surgery interval^a^	Prior MRI	Side of Lesion	MRI assessment	Biopsy Method	Pathology	MRI finding	Mammographic density	Mammography assessment
35–40	60.0	Yes	Contralateral	BI-RADS 3	US	IDC	Mass	Heterogeneously dense	BI-RADS 1
50–55	56.3	Yes	Contralateral	BI-RADS 4	US	DCIS	Nonmass	Heterogeneously dense	BI-RADS 2
45–50	24.7	Yes	Contralateral	BI-RADS 4	US	Mucinous carcinoma	Nonmass	Heterogeneously dense	BI-RADS 2
35–40	38.7	Yes	Contralateral	BI-RADS 4	MRI	ILC	Nonmass	Heterogeneously dense	BI-RADS 2

*IDC* invasive ductal carcinoma, *DCIS* ductal carcinoma in situ, *ILC* invasive lobular carcinoma

^a^Interval between initial surgery and screening breast MR examination by which the subsequent cancer was detected (months)

Among the 8 examinations with suspicious extramammary findings, five were finally considered negative based on image-guided biopsy (*n* = 3) or further imaging evaluation (PET-CT, whole body bone scan) (*n* = 2) with no evidence of malignancy for more than 1 year. Of the three examinations with extramammary cancer, two were histologically confirmed by US-guided biopsy (*n* = 1, chest wall) or surgical excision (n = 1, mediastinal LN). The remaining one patient was diagnosed with sternum metastasis based on imaging alone, which was initially detected on breast MRI and subsequently confirmed by whole body bone scan and PET-CT (Table 4). Therefore, the overall cancer detection rate for MRI was 6.7 per 1000 examinations (7 of 1053).

**Table 4:** Pathological characteristics of MR-detected intramammary breast cancers and extramammary cancers at surveillance breast MR imaging in women with a personal history of breast cancer

					Subsequent cancer						Initial primary breast cancer					
Age range, years	Location	Final assessment	Type of surgery	Initial surgery interval^a^	Pathology	Size, mm	TNM	ER	PR	HER2	Pathology	Size, mm	TNM	ER	PR	HER2
35–40	contralateral breast	BI-RADS 3	Mastectomy	60.0	IDC	9	T1bN0M0	pos	pos	neg	IDC	30	T2N1M0	neg	neg	neg
50–55	contralateral breast	BI-RADS 4	BCS	56.3	DCIS	15	TisN0M0	pos	pos	neg	DCIS	15	TisN0M0	pos	pos	neg
45–50	contralateral breast	BI-RADS 4	BCS	24.7	Mucinous carcinoma	7	T1bN0M0	pos	pos	neg	DCIS with microinvasion	1	T1miN0M0	pos	pos	neg
40–45	contralateral breast	BI-RADS 4	BCS	38.7	ILC	11	T1cN0M0	pos	pos	neg	IDC	26	T2N0M0	pos	pos	neg
70–75	sternum	Suspicious for malignancy	BCS	81.5	N/A	–	–	–	–	–	IDC	23	T2N0M0	pos	neg	neg
60–65	mediastinum	Suspicious for malignancy	BCS	60.4	Metastatic carcinoma	–	–	pos	neg	neg	ILC	26	T2N0M0	pos	neg	neg
45–50	chest wall	Suspicious for malignancy	Mastectomy	60.4	Metastatic carcinoma	–	–	pos	neg	neg	IDC	10	T1bN0M0	pos	pos	neg

*BCS* breast conservation surgery, *IDC* invasive ductal carcinoma, *DCIS* ductal carcinoma in situ, *ILC* invasive lobular carcinoma, *N/A* not available

^a^Interval between initial surgery and surveillance breast MR examination by which the subsequent cancer was detected (months)

### Cancer detection yield for Intramammary lesions

The abnormal interpretation rate for MR-detected intramammary lesions was 7.2% (76 of 1053) and the cancer detection rate for intramammary lesions was 3.8 per 1000 examinations (4 of 1053). The PPV_1_ was 5.3% (4 of 76) and PPV_3_ was 15.8% (3 of 19). There was only one false-negative result during the study period, corresponding to the aforementioned invasive ductal carcinoma assigned as category 3. The sensitivity of surveillance MR imaging was 75.0% (3 of 4 [95% confidence interval: 71.0%, 79.0%]) and the specificity was 98.3% (1031 of 1049 [95% confidence interval: 97.1%, 99.5%]).

### Cancer detection yield according to interval between initial surgery and MRI

The overall cancer detection rate for MRI was significantly greater in MR examinations performed with more than a 36-month interval following initial surgery than those performed at or less than a 36-month interval (17.4 per 1000 examinations vs. 1.4 per 1000 examinations, *p* = 0.006). None of the other performance statistics showed a significant difference between the two groups (Table 2).

The mean interval between prior US and MR examinations was slightly greater in MR examinations performed ≤36 months than those performed > 36 months following initial surgery (6.3 ± 1.0 months vs. 5.9 months±1.6 months, *p* < 0.001), but with a mean difference of 0.4 months.

## Discussion

With recognition of the increased future breast cancer risk in patients with a personal history of treated breast cancer and the decreased sensitivity of mammography in dense breasts, several studies have recently investigated the performance of surveillance breast MRI examinations [15, 19–23]. Although the patient population and study design differ somewhat between studies, the reported cancer detection rates range from 10.0 to 18.1 per 1000 examinations [15, 19, 20, 22, 23], which are higher than the overall cancer detection rate in our study. One possible explanation is that 37.9% of our study population underwent mastectomy, whereas the majority of patients underwent breast conservation surgery in most studies [11, 15, 22]. Another possible explanation is that the majority (88.1%) of our study population had previously undergone preoperative breast MR imaging, whereas only 38.9% and 54.2% of the study population in the study of Brennan et al. and Lehman et al. had baseline preoperative MR examinations, respectively [19, 22]. In addition, our study population had received routine supplemental surveillance US, with a median interval of 6.1 months between prior surveillance US and MR imaging. All of the four MR-detected intramammary cancers in our study were not detected by previous surveillance US performed prior to MR imaging. Therefore, the MR-detected cancers in our study are more likely to represent truly newly developed cancers after treatment of initial breast cancer, which may be difficult to detect with surveillance US.

The fact that the cancer detection rate for MR imaging performed at more than 3 years after surgery (17.4 per 1000) was greater than that for examinations performed within 3 years (1.41 per 1000) may provide a basis for establishing guidelines regarding timing of surveillance MR imaging initiation following definitive breast cancer surgery. Our overall cancer detection rate was similar to the incidence cancer detection rate of screening breast MR imaging in average risk women in a recent study (7.5 per 1000 examinations, 13 of 1741) [24]. In another study on women with a history of breast conservation therapy, of whom 91.8% underwent preoperative MR imaging and all had undergone supplemental surveillance US, a more than 24-month interval between initial surgery and MR imaging was an independent factor associated with MR-detected cancers [15]. Similar results have been reported for breast MR imaging screening of women with average risk of breast cancer, with no screening-detected breast cancer diagnoses made until almost 3 years after a negative MR study [24]. This has important implications for the effective implementation of breast MR imaging as a surveillance modality in the future, as breast MR imaging early in the post-treatment surveillance period may have relatively low cancer yields − especially with the increased use of preoperative breast MR imaging.

The overall abnormal interpretation rate (8.0%) in our study was slightly lower than prior studies, which ranged from 10.7% to 19.3% [15, 20, 22, 25]. Although the PPV_1_ (5.3%) was slightly lower than that in previous MR imaging studies (approximately 9.4%) [15, 20], it was still higher than mammographic screening benchmarks from 2004 to 2008 according to the Breast Cancer Surveillance Consortium (4.3%) [26]. In addition, the PPV_3_ in our study for intramammary lesions, 15.8% (3 of 19), was higher than the lower range of reported PPV values of surveillance US in women with a personal history of breast cancer, which ranged from 9.4% to 52.6% [27–30]. Reported PPV values of surveillance breast MR imaging in women with a personal history of breast cancer have been consistently similar to or higher than that of surveillance US [27–30]. Furthermore, surveillance MR imaging detected three extramammary rcancers (0.3%, 3 of 1053), which accounted for 42.8% of MR-detected cancers. Therefore, breast MR imaging may be more advantageous compared to US as an adjunctive surveillance tool, considering its low abnormal interpretation rate and ability to detect extramammary malignancy.

Our study had several limitations. First, this was a retrospective study from a single institution. Although our institution recently implemented breast MRI imaging into our post-treatment surveillance protocol to be performed two and five years after surgery, MR imaging was also performed at the request of clinicians or patients and therefore, the intervals between surgery and MR imaging were variable. Second, patients underwent surveillance with mammography and US prior to MR imaging, which could have affected the true cancer yield of MRI. Third, the median interval between initial breast cancer surgery and first-time surveillance MR examination (30.1 months, range, 12.1–240.2 months) was relatively short.

## Conclusions

Our data suggest that surveillance breast MR imaging may be considered in women with a history of breast cancer, considering the low abnormal interpretation rate and its high diagnostic performance. However, the cancer detection rate was low and implementation may be more effective after more than 3 years after surgery. Further research on the appropriate timing for surveillance breast MR imaging initiation is required, especially in patients who have undergone preoperative breast MR imaging and supplemental surveillance US.

## Abbreviations

BCS: Breast conserving surgery
BI-RADS: Breast Imaging Reporting and Data System
CDR: Cancer detection rate
MR: Magnetic resonance
PP: Positive predictive value
US: Ultrasound
